# Transcriptomic Analysis Identifies Acrolein Exposure-Related Pathways and Constructs a Prognostic Model in Oral Squamous Cell Carcinoma

**DOI:** 10.3390/ijms27020632

**Published:** 2026-01-08

**Authors:** Yiting Feng, Lijuan Lou, Liangliang Ren

**Affiliations:** 1 State Key Laboratory of Medical Proteomics, National Center for Protein Sciences (Beijing), Beijing Institute of Lifeomics, Beijing 102206, China; fengyiting28@163.com (Y.F.);; 2 Department of Biochemistry and Molecular Biology, School of Basic Medical Sciences, Anhui Medical University, Hefei 230032, China

**Keywords:** acrolein, OSCC, environmental pollutants, bioinformatics, machine learning, prognostic model

## Abstract

Acrolein, a highly reactive environmental toxicant widely present in urban air and tobacco smoke, has been implicated in the development of multiple malignancies. In oral tissues, chronic acrolein exposure induces oxidative stress, inflammation, and genetic mutations, all of which are closely linked to the development of oral squamous cell carcinoma (OSCC). Although accumulating evidence indicates a strong association between acrolein exposure and OSCC, its prognostic significance remains poorly understood. In this study, we analyzed transcriptome data to identify differentially expressed genes (DEGs) between tumor and adjacent normal tissues, and screened acrolein-related candidates by intersecting DEGs with previously identified acrolein-associated gene sets. Functional alterations of these genes were assessed using Gene Set Variation Analysis (GSVA), and a protein–protein interaction (PPI) network was constructed to identify key regulatory genes. A prognostic model was developed using Support Vector Machine–Recursive Feature Elimination (SVM-RFE) combined with LASSO-Cox regression and validated in an independent external cohort. Among the acrolein-related DEGs, four key genes (PLK1, AURKA, CTLA4, and PPARG) were ultimately selected for model construction. Kaplan–Meier analysis showed significantly worse overall survival in the high-risk group (*p* < 0.0001). Receiver operating characteristic (ROC) curve analysis further confirmed the strong predictive performance of the model, with area under the curve (AUC) values of 0.72 at 1 year, 0.72 at 3 years, and 0.75 at 5 years. Furthermore, the high risk score was significantly correlated with a ‘cold’ immune microenviroment, suggesting that acrolein-related genes may modulate the tumor immune microenvironment. Collectively, these findings highlight the role of acrolein in OSCC progression, suggesting the importance of reducing acrolein exposure for cancer prevention and public health, and call for increased attention to the relationship between environmental toxicants and disease initiation, providing a scientific basis for public health interventions and cancer prevention strategies.

## 1. Introduction

Acrolein is a highly reactive α,β-unsaturated aldehyde and a ubiquitous environmental pollutant widely present in industrial emissions, agricultural activities, and various combustion processes, including vehicle exhaust and tobacco smoke [1,2]. Classified as “probably carcinogenic to humans” (Group 2A) by the IARC [3], acrolein exhibits strong electrophilic activity, forming DNA and protein adducts that trigger oxidative stress, inflammation, and genetic mutations [4,5,6]. These pathological processes are closely linked to tumorigenesis, and emerging evidence specifically implicates acrolein in the pathogenesis of respiratory and oral disorders [7,8,9,10].

Oral squamous cell carcinoma (OSCC) represents approximately 50% of all head and neck malignancies and is characterized by aggressive biological behavior and a five-year survival rate of only 50% [11,12]. While tobacco use is a primary etiological factor [13,14], studies indicate that whereas nicotine drives addiction, acrolein serves as a predominant contributor to tobacco smoke carcinogenicity [9,15]. In oral tissues, chronic acrolein exposure induces epithelial damage and genomic instability, potentially synergizing with other risk factors like betel nut chewing to promote OSCC progression [16,17]. Despite its established toxicity, the specific prognostic relevance of acrolein-related gene expression in OSCC has not been systematically investigated.

Although previous studies have examined the toxicity and molecular mechanisms of acrolein, its prognostic relevance in OSCC has not been systematically investigated. To date, no studies have integrated acrolein-related gene expression with OSCC clinical outcome data to construct a robust prognostic model. Advances in high-throughput sequencing and bioinformatics now enable the identification of disease-associated gene signatures and the development of predictive models using machine-learning and survival-analysis approaches, such as support vector machine–recursive feature elimination (SVM-RFE) and LASSO-Cox regression [18,19]. These analytical strategies provide powerful tools for discovering functionally relevant biomarkers and establishing precise prognostic systems.

In this study, we comprehensively analyzed OSCC transcriptomic data from The Cancer Genome Atlas (TCGA) to identify differentially expressed acrolein-related genes. We constructed prognostic risk models using protein—protein interaction (PPI) network analysis, machine-learning algorithms, and validated the models using independent datasets from the Gene Expression Omnibus (GEO). Additionally, we evaluated the relationships between the prognostic model, immune-cell infiltration patterns, and molecular pathways. Our findings not only elucidate the functional roles of acrolein-related genes in OSCC but also provide a novel framework for risk stratification and precision oncology.

Therefore, this study aimed to explore the potential association between acrolein and OSCC patient prognosis by constructing an acrolein-related prognostic model. The work provides a theoretical basis for risk assessment, personalized treatment, and public health strategies for OSCC, while also contributing to the investigation of environmental pollutants relevant to human OSCC.

## 2. Results

### 2.1. Identification of Acrolein-Related Differentially Expressed Genes and Pathway Enrichment Analysis in OSCC

**Figure 1:**
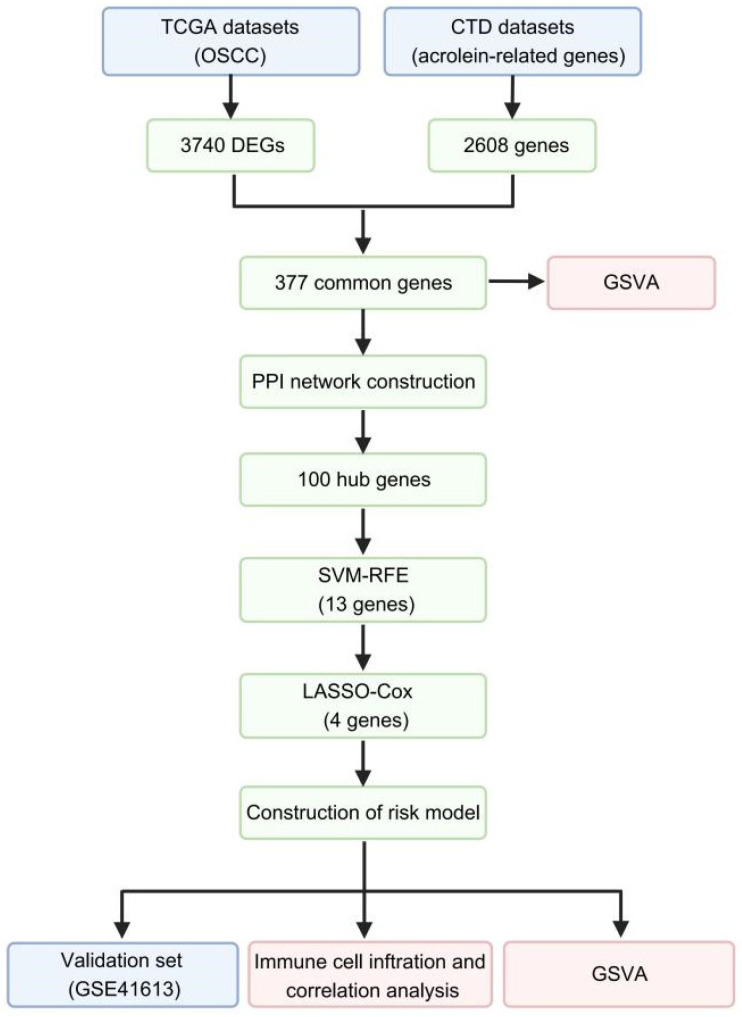
Flow chart of the study.

**Figure 2:**
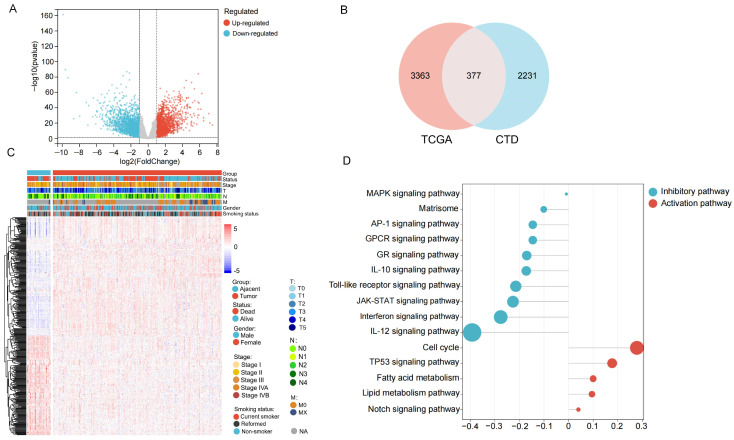
Identification of acrolein-related differentially expressed genes and GSVA pathway enrichment analysis in OSCC. (**A**) Volcano plot illustrating the differential gene expression profiles comparing tumor samples with adjacent normal tissues within the TCGA cohort; (**B**) Venn diagram illustrating the overlap between DEGs from TCGA and acrolein-related genes from the CTD; (**C**) Heatmap depicting expression levels of the intersecting genes across samples; (**D**) GSVA pathway enrichment analysis of acrolein-related DEGs in OSCC.

In the present study, transcriptomic profiles from OSCC patients within the TCGA-HNSC cohort were designated as the primary training set. Conversely, the GSE41613 dataset functioned as an independent external validation cohort. The overall analytical workflow is presented in Figure 1. Following data preprocessing and normalization, a total of 290 tumor specimens and 40 adjacent normal tissue samples from the TCGA cohort were retained for subsequent analysis (Supplementary Table S1). A total of 3740 differentially expressed genes (DEGs) were identified, and the results were visualized using a volcano plot (Figure 2A).

In parallel, 2608 acrolein-related genes were retrieved from the Comparative Toxicogenomics Database (CTD). By intersecting these genes with the DEGs identified in OSCC, 377 overlapping genes were obtained (Figure 2B) (Supplementary Table S2). The expression patterns of these intersected genes across samples were visualized using a heatmap (Figure 2C). Subsequently, the 377 intersecting genes underwent pathway enrichment analysis to quantify specific enrichment levels. Differential analysis was thereafter employed to discern pathways exhibiting statistical significance (*p* < 0.05), the results of which are visualized in Figure 2D.

Pathway enrichment analysis identified 15 significantly dysregulated signaling pathways in OSCC. Notably, pathways related to the cell cycle, NOTCH signaling, TP53 signaling, and fatty acid metabolism were activated, whereas JAK-STAT signaling, interferon signaling, Toll-like receptor signaling, and MAPK signaling were suppressed. These results implicate acrolein-associated genes in the pathogenesis of OSCC, potentially through the dysregulation of critical signaling cascades—such as NOTCH, TP53, and JAK-STAT—and metabolic circuits, consequently impacting oncogenesis and cellular equilibrium.

### 2.2. Construction of the PPI Network and Identification of Hub Genes

**Figure 3:**
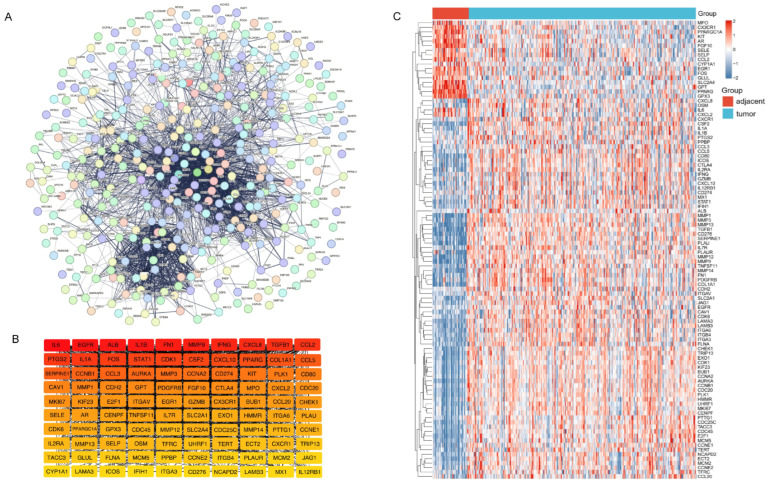
Construction of the PPI network and identification of hub genes. (**A**) Protein–protein interaction (PPI) network constructed based on the intersecting genes; (**B**) Hub genes identified using the Degree algorithm in the CytoHubba plugin; (**C**) Heatmap depicting expression patterns of the hub genes across samples.

The 377 intersecting genes were mapped via the STRING database to generate a PPI network, which yielded a complex structure comprising 348 nodes and 6078 edges (Figure 3A). Subsequently, the network topology was analyzed using the CytoHubba plugin in Cytoscape (V3.10.2), where gene importance was ranked based on the Degree algorithm. This process prioritized 100 hub genes (Figure 3B), whose expression landscapes across samples were depicted in a heatmap (Figure 3C).

### 2.3. Construction of an Acrolein-Related Prognostic Risk Model in OSCC

**Figure 4:**
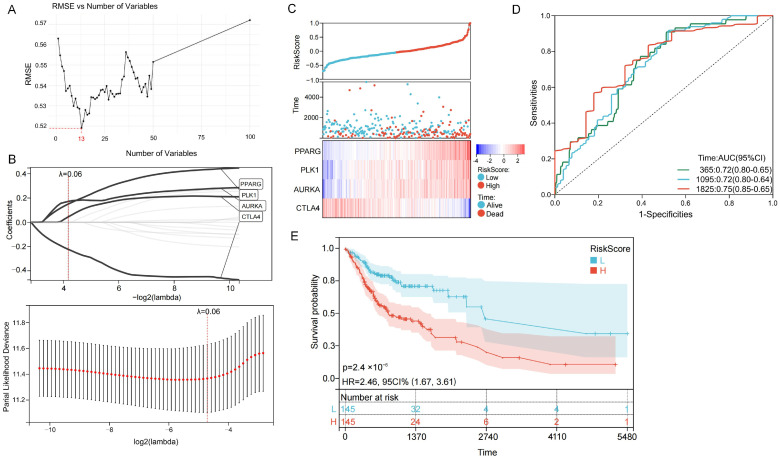
Construction of the acrolein-related prognostic risk model using machine learning. (**A**) Biomarkers selected by the SVM-RFE algorithm; (**B**) Prognostic risk model constructed using LASSO-Cox regression analysis; (**C**) Relationships between patient survival status and mRNA expression levels of the four genes with risk scores; (**D**) ROC curves for predicting 1-, 3-, and 5-year overall survival based on the risk scores; (**E**): Kaplan–Meier survival curves comparing high-risk and low-risk patient groups.

The SVM-RFE algorithm was initially applied to the 100 hub genes to select candidate features, resulting in 13 feature genes (Figure 4A). Subsequently, LASSO-Cox regression analysis was conducted to further refine the feature set, yielding a prognostic risk model comprising four genes (Figure 4B). The risk score for each patient was calculated using the following formula:

Riskscore = 0.1719 × PLK1 + 0.0444 × AURKA − 0.2229 × CTLA4 + 0.1502 × PPARG

Using the median risk score as a cutoff, the patient cohort was dichotomized into high- and low-risk groups (Supplementary Table S3). We subsequently investigated the interplay between risk scores, survival outcomes, and the expression profiles of the four signature genes. As depicted in Figure 4C, an elevated risk score was significantly correlated with diminished survival time; conversely, the mRNA expression levels of the four genes displayed a positive trajectory parallel to the rising risk scores.

The predictive performance of the risk model was evaluated using the area under the receiver operating characteristic (ROC) curve (AUC), with values of 0.72, 0.72, and 0.75 for 1-, 3-, and 5-year overall survival (OS), respectively (Figure 4D). To further quantify the model’s discriminatory power, the Concordance Index (C-index) was calculated, yielding a value of 0.73 (95% CI: 0.68–0.78), which indicates robust predictive capability. Kaplan–Meier survival analysis demonstrated that patients in the low-risk group had significantly better OS compared with those in the high-risk group (*p* < 0.001) (Figure 4E). The 95% confidence interval (CI) for the hazard ratio (HR) was 1.63–3.57, confirming that the high-risk group is associated with a lower survival rate.

### 2.4. Validation of the Prognostic Risk Model Using the GSE41613 Dataset

**Figure 5:**
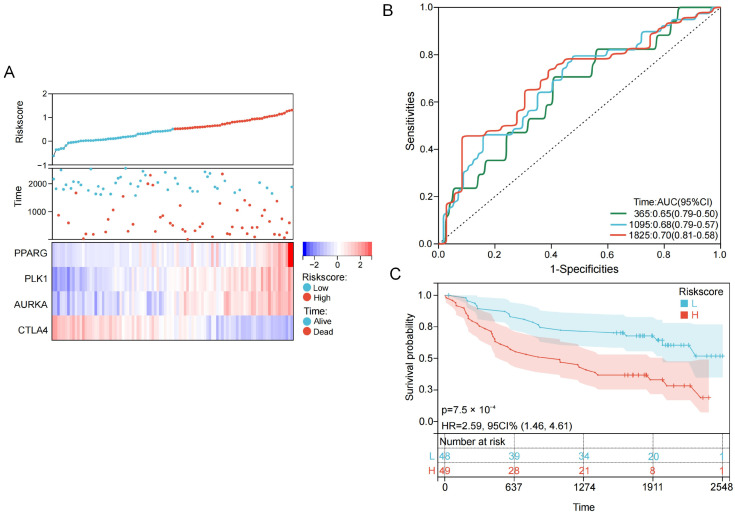
Validation of the acrolein-related prognostic risk model using the GSE41613 cohort. (**A**) Relationships between patient survival status and mRNA expression levels of the four genes with increasing risk scores in the GSE41613 validation cohort; (**B**) ROC curves for predicting 1-, 3-, and 5-year overall survival based on risk scores in the validation cohort; (**C**) Kaplan–Meier survival curves comparing high-risk and low-risk patient groups in the validation cohort.

To validate the prognostic performance of the risk model, the risk score model derived from the TCGA dataset was applied to the GSE41613 cohort, which included 97 patients. Risk scores were calculated for all patients in the validation set, and higher risk scores were significantly associated with shorter survival rate. The mRNA expression patterns of the four genes were consistent with those observed in the TCGA dataset (Figure 5A).

The predictive performance of the risk model was further evaluated using the area under the ROC curve, yielding values of 0.65, 0.68, and 0.70 for 1-, 3-, and 5-year overall survival, respectively. To further validate the model’s discrimination, the Concordance Index (C-index) was calculated as 0.68 (95% CI: 0.63–0.73). Kaplan–Meier survival analysis revealed a distinct prognostic disparity between the risk strata, with patients in the high-risk category experiencing significantly reduced overall survival (Figure 5B,C). The Hazard Ratio (HR), with a 95% confidence interval (CI) of 1.46–4.61, substantiated the elevated mortality risk associated with the high-risk group relative to the low-risk group. Furthermore, a *p*-value < 0.05 corroborated the statistical divergence of the survival trajectories. Collectively, these findings underscore the robust predictive precision and stability of the constructed model across both training and validation cohorts.

### 2.5. Clinical Characteristics Analysis and GSVA Pathway Enrichment Analysis Between High- and Low-Risk Patients

**Figure 6:**
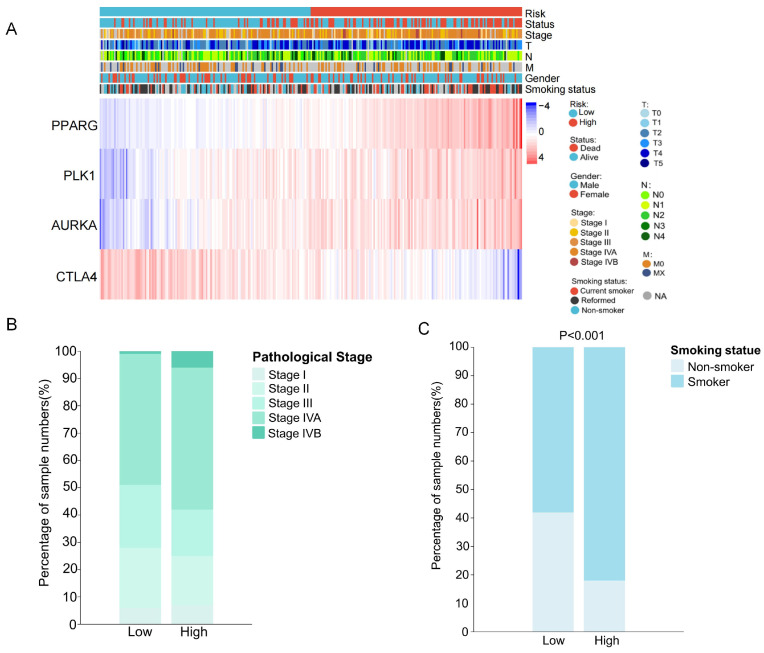
Clinical characteristics and pathway enrichment analysis between high- and low-risk patients. (**A**) Heatmap showing the expression levels of the four genes in relation to clinical features in the TCGA dataset; (**B**) Bar plot illustrating the results of chi-square tests for smoking status; (**C**) Bar plot depicting the results of chi-square tests for different pathological stages.

To investigate the relationship between the acrolein-related OSCC prognostic risk model and clinical characteristics, pathological stage, TNM stage, sex, smoking status, and survival status were compared in the training dataset (Figure 6A). Chi-square tests were applied to assess differences in pathological stage between high- and low-risk patients. Although the proportion of high-risk patients appeared higher in stage IVB, the association between risk groups and pathological stage was not statistically significant (χ^2^ = 4.76, *p* = 0.3132) (Figure 6B). To explicitly validate the specific prognostic value of the signature in the context of acrolein exposure, we performed a stratified analysis within the smoker subgroup. We applied a chi-square test to evaluate the association between the model’s risk stratification and patient survival status specifically among smokers (Figure 6C). The analysis revealed a highly significant correlation (x^2^ = 14.22, *p* < 0.001), indicating that the high-risk group was strongly associated with poor clinical outcomes in patients with a history of smoking. This compelling statistical evidence confirms that the 4-gene signature effectively captures biologically relevant risks driven by environmental acrolein exposure.

**Figure 7:**
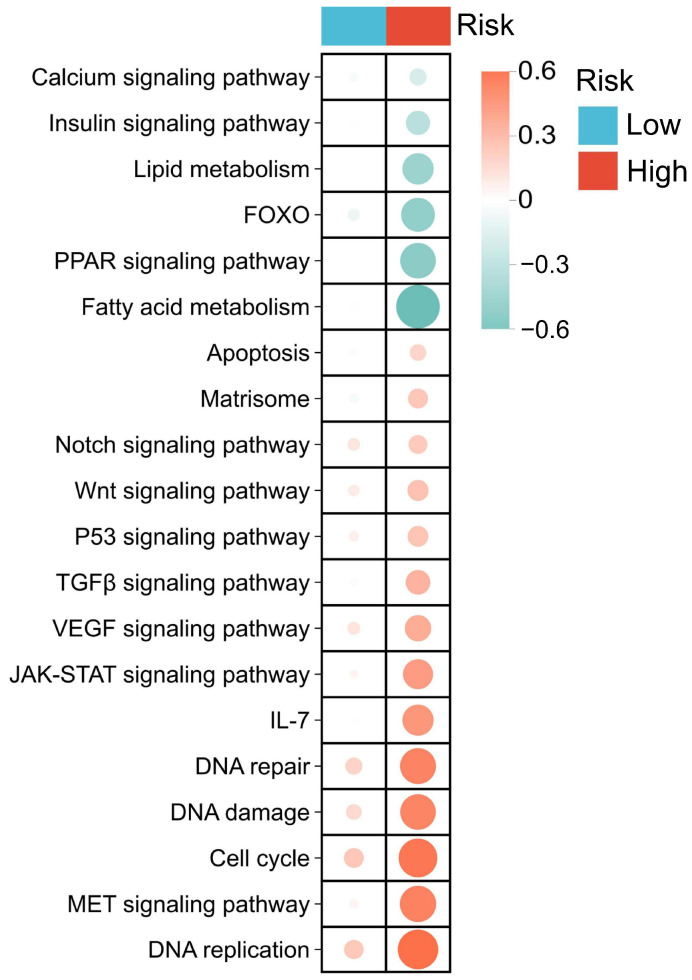
GSVA pathway enrichment analysis between high- and low-risk patients.

Furthermore, GSVA pathway enrichment analysis was performed on acrolein-related differentially expressed genes stratified by risk groups, identifying 20 significantly enriched pathways (Supplementary Table S4). The results revealed activation of the Notch, WNT, p53, TGF-β, and JAK-STAT signaling pathways, whereas the PPAR signaling pathway, calcium signaling, and fatty acid metabolism pathways were suppressed. These findings indicate that acrolein-related differentially expressed genes play a critical role in the biological effects of acrolein and the development of OSCC. Moreover, compared with the low-risk group, the activation or suppression of these pathways was more pronounced in the high-risk group, suggesting that the prognostic risk model exhibits robust predictive capability (Figure 7).

### 2.6. Distinct Immune Infiltration Patterns in Risk Groups and Escape Mechanisms Driven by the 4-Gene Signature

**Figure 8:**
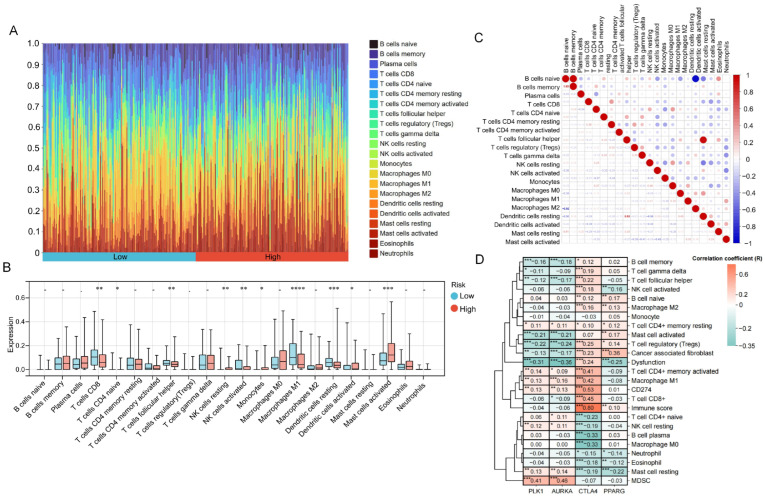
Distinct Immune Infiltration Patterns in Risk Groups and Escape Mechanisms Driven by the 4-Gene Signature. (**A**) Immune cell infiltration profiles in high- and low-risk patient groups; (**B**) Differential abundance of immune cells between high- and low-risk patients; (**C**) Correlation heatmap of 22 immune cell types. Statistical significance: * *p* < 0.05, ** *p* < 0.01, *** *p* < 0.001, **** *p* < 0.0001; (**D**) Correlation heatmap between the 4-gene signature and immune features. Statistical significance: * *p* < 0.05, ** *p* < 0.01, *** *p* < 0.001.

Immune infiltration analysis of high- and low-risk patient samples was performed using the CIBERSORT algorithm (Figure 8A). Comparative analysis of immune cell composition between high- and low-risk OSCC patients revealed distinct patterns. In the high-risk group, resting NK cells, monocytes, activated dendritic cells, and activated mast cells showed a significant higher trend compared with the low-risk group, with activated mast cells exhibiting the most pronounced increase (*p* < 0.001). In contrast, CD8+ T cells, NK cells activated, macrophage M1 showed a lower trend in high-risk group, among which M1 macrophages showed the most significant increase (*p* < 0.0001) (Figure 8B).

The correlation heatmap of 22 immune cell types revealed that memory B cells and B cells naive, as well as resting mast cells and regulatory T cells (Tregs), were highly positively correlated, with correlation coefficients of 1.0 and 0.93, respectively. In contrast, activated dendritic cells and B cells naive exhibited a strong negative correlation, with a coefficient of −0.92 (Figure 8C).

Furthermore, to elucidate the deep biological integration between the 4-gene signature and OSCC immune-escape mechanisms, we performed a further analysis correlating gene expression with tumor-infiltrating immune cells and TIDE functional scores (Figure 8D). This analysis revealed the escape pathways driven by the signature components: the CTLA4 component correlated with CD8+ T cells (R = 0.45) and the overall Immune Score (R = 0.80), indicating the CTLA4 as a proxy for T cell infiltration. In addition, the CIBERSORT findings regarding elevated mast cells in high-risk patients, whereas the PPARG showed a specific affinity for cancer associated fibroblasts (R = 0.36) and macrophage M2, and was the unique driver associated with activated mast cells (R = 0.17), suggesting a stromal-mediated immune exclusion mechanism; In contrast, the proliferation-related genes PLK1 and AURKA were primarily associated with MDSC recruitment (R > 0.40).

## 3. Discussion

Acrolein, a highly reactive α,β-unsaturated aldehyde, is an environmental pollutant with mutagenic and genotoxic properties [20]. Accumulating evidence suggests that acrolein plays a critical role in carcinogenesis via multiple mechanisms, including DNA adduct formation, induction of oxidative stress, inflammatory responses, and disruption of cellular signaling pathways [21,22,23,24]. In oral tissues, chronic exposure to acrolein has been reported to induce epithelial damage, genomic instability, and pro-tumorigenic microenvironmental changes, all closely linked to the initiation and progression of OSCC [25]. Epidemiological evidence has shown the association between environmental or tobacco-related acrolein exposure and elevated OSCC risk, emphasizing its role as a potential etiological factor [26]. Despite these insights, few studies have systematically investigated the prognostic significance of acrolein-related molecular alterations in OSCC.

Through gene set variation analysis (GSVA) of acrolein-related OSCC genes, we observed significant activation of cell cycle, p53, and Notch signaling pathways, whereas JAK-STAT, MAPK, and Toll-like receptor (TLR) pathways exhibited inhibitory trends. The activation of cell cycle and p53 pathways is consistent with previous reports, suggesting that acrolein might be associated with DNA damage and genomic instability, potentially leading to the activation of cell cycle checkpoints and p53-mediated responses, which could ultimately influence the proliferation and apoptosis of oral epithelial cells [8,27,28,29].

On the other hand, JAK-STAT, MAPK, and TLR pathways have been reported to be influenced by acrolein exposure as well as in OSCC. Previous studies have shown that acrolein can interfere with MAPK (e.g., JNK/p38), NF-κB, and IRF3 signaling by suppressing immune cell responses, such as those of macrophages, thereby inhibiting pro-inflammatory responses and immune activity [30,31,32]. Simultaneously, these pathways are also involved in tumor proliferation, immune evasion, and microenvironmental regulation in OSCC [33,34,35]. Our observation of inhibitory trends in these pathways aligns with the hypothesis that acrolein may exert immunosuppressive effects within the tumor microenvironment.

Notch signaling has been extensively reported to contribute to tumor proliferation, invasion, and poor prognosis in OSCC, for instance by promoting epithelial–mesenchymal transition (EMT) and migration [36]. However, to date, there is no evidence that acrolein directly modulates Notch signaling. Therefore, our findings imply that acrolein might regulate Notch activity in OSCC via as-yet-uncharacterized mechanisms, providing novel molecular insights into its carcinogenic effects. Taken together, our results not only validate the classical oncogenic mechanisms in OSCC and propose a new hypothesis: acrolein exposure may contribute to OSCC development potentially by modulating Notch signaling while concurrently associating with the suppression of immune- and inflammation-related pathways to modulate the tumor microenvironment. These findings deepen our understanding of the molecular mechanisms by which acrolein, as an environmental carcinogen, might contribute to OSCC, and provide a theoretical basis for molecular prognostic assessment and environmental intervention strategies in this malignancy.

In this study, we systematically investigated the acrolein-related DEGs in OSCC and developed a prognostic model consisting of four key genes: PLK1, AURKA, CTLA4, and PPARG. This gene signature effectively stratified patients into high- and low-risk groups, which displayed significantly distinct overall survival outcomes. Its predictive performance was consistently validated in an independent GEO cohort.

Beyond statistical selection, the biological relevance of the signature genes to acrolein and tobacco smoke exposure is substantiated by retrospective evidence validation. Studies report that PLK1 inhibition decreases mutational activity in epithelial cells exposed to tobacco carcinogens [37], while AURKA demonstrates a significant gene-environment interaction where its carcinogenic risk is synergistically modulated by smoking status specifically in OSCC [38]. These findings suggest that acrolein exposure induces genomic instability, activating PLK1 and AURKA signaling to facilitate uncontrolled proliferation. Functionally, PLK1 overexpression suppresses apoptosis and promotes chromosomal instability [39], whereas AURKA drives centrosome amplification and aberrant mitosis, increasing metastatic potential [39]. Both PLK1 and AURKA were reported overexpressed in OSCC cells (Figure S8), and DepMap analysis revealed a high dependency on these genes for OSCC cell survival (Figure S7). Furthermore, our immune analysis revealed a critical escape mechanism: PLK1 and AURKA were significantly correlated with MDSC (myeloid-derived suppressor cell) recruitment (Figure 8D). This suggests that high-grade, proliferative tumors not only grow rapidly but actively exclude effector cells via myeloid-mediated suppression, creating a hostile microenvironment.

Regarding PPARG, we identified a dual link to both etiology and disease progression. Etiologically, cigarette smoke exposure (a primary source of acrolein) has been confirmed to functionally suppress PPARG activity [40], which is consistent with the suppression of PPAR signaling in our high-risk group (Figure 7). However, PPARG expression was significantly elevated in the high-risk group (Figures S5 and S12). Our multi-dimensional analysis resolves this conflicts by highlighting stromal contributions. PPARG expression was strongly positively correlated with stromal score and cancer-associated fibroblasts (CAFs), but negatively correlated with tumor purity (Figure 8D and Figure S4). This indicates that the elevated PPARG signal detected in high-risk patients could predominantly originate from the infiltrating stromal compartment rather than the tumor cells (Figure S9). Moreover, we identified PPARG to be significantly associated with mast cell activation (Figure 8D), aligning with evidence that acrolein is a potent activator of mast cells [41]. The accumulation of CAFs and activated mast cells likely creates a physical and inflammatory “stromal barrier” that excludes anti-tumor immunity. Therefore, high PPARG expression serves as a robust proxy for a stromal-rich, invasive microenvironment, explaining its association with poor clinical outcomes.

Interestingly, our model identified CTLA4 as a protective factor in OSCC, with lower expression observed in the high-risk group. While this trend contrasts with its canonical role as an immune checkpoint, our immune deconvolution analysis provides a clear mechanistic rationale for this observation (Figure 8D and Figure S6). We found that CTLA4 expression was strongly positively correlated with the immune score and CD8+ T-cell infiltration, and the tumor cells barely express CTLA4 (Figures S2 and S3). This implies that in the context of bulk RNA-seq, CTLA4 levels function primarily as a surrogate marker for the density of tumor-infiltrating lymphocytes rather than reflecting tumor-intrinsic signaling alone. Therefore, high CTLA4 expression signals an immunologically active (‘hot’) tumor microenvironment, in which the survival benefit by the cytotoxic T cells outweighs the checkpoint’s suppressive effects. Conversely, low CTLA4 expression in the high-risk group reflects an acrolein-associated “immune-cold” microenvironment, characterized by T-cell exclusion and a lack of immune surveillance, which is fundamentally associated with poor prognosis (Figure 8B and Figure S1B).

Compared with previously reported OSCC prognostic models based on genome-wide screening or traditional clinicopathological features (e.g., TNM staging), this study is the first to construct a prognostic model centered on acrolein-related genes mechanistically linked to carcinogenesis. GSVA revealed significant activation or suppression of Notch, Wnt, TGF-β, p53, PPAR, and lipid metabolism pathways in high-risk patients, consistent with aggressive OSCC molecular mechanisms (Figure 7). To rigorously validate our signature and circumvent the limitations of standard in vitro models, we implemented a ‘multidimensional validation’ strategy. We investigated the HPA immunohistochemistry and DepMap CRISPR screenings to confirm the tumor-intrinsic essentiality of PLK1 and AURKA, while utilizing the TISCH single-cell dataset to validate the microenvironmental specificity of CTLA4 (T-cells) and PPARG (CAFs/Macrophages) (Figure S9). This multi-layered approach confirms that the signature captures the complete tumor ecosystem---intrinsic malignancy, stromal remodeling and immune exclusion.

The translational value of this study extends beyond statistical significance to informing clinical management: (1) Risk-Adapted Surveillance: Supported by the DCA results, we propose that high-risk patients require intensified post-operative surveillance (e.g., shortened follow-up intervals and frequent imaging) to detect early recurrence. Conversely, low-risk patients identified by our model might be spared from excessive overtreatment, improving quality of life (Figures S10 and S11). (2) Environmental-Based Intervention: Unlike generic prognostic models, our signature is grounded in environmental toxicology. This provides specific utility for lifestyle intervention: the model identifies patients whose poor prognosis is driven by environmental toxicity pathways (acrolein response). This reinforces the clinical necessity for strict smoking cessation and environmental exposure reduction as a critical part of the disease management plan for high-risk individuals. (3) Guiding Precision Therapy: Our analysis offers a molecular rationale for targeted drug selection in high-risk patients: (i) Targeting Mitotic Kinases: The hallmark upregulation of PLK1 and AURKA in the high-risk group suggests these patients are ideal candidates for small-molecule inhibitors (e.g., Volasertib or Alisertib) currently in clinical trials. (ii) Immunotherapy Optimization: The hallmark immune exclusion (Low CTLA4/CD8+) suggests that high-risk patients are poor candidates for single-agent PD-1 blockade but may benefit from strategies that “prime” the immune microenvironment. (iii) Stromal Modulation (PPARG Antagonists): Addressing the PPARG-driven stromal barrier, we propose a combinatorial strategy. Although PPARG is often targeted with agonists in metabolic diseases, our data indicates that high PPARG is associated with the aggressive, fibroblast-rich phenotype in OSCC. Therefore, we suggest investigating PPARG antagonists (e.g., GW9662, as supported by recent OSCC preclinical studies) to disrupt this tumor-stromal crosstalk. This blockade might “normalize” the immunosuppressive stroma, potentially sensitizing high-risk tumors to subsequent T-cell based immunotherapies.

However, we must acknowledge specific limitations regarding our data sources. The initial pool of acrolein-related genes was retrieved from public toxicogenomics databases. To mitigate potential noise, we employed rigorous multi-step statistical screening and external validation to serve as a robust biological filter. Nevertheless, direct mechanistic validation was not performed in this prognostic-focused study. Future experimental studies are required to elucidate the precise molecular pathways. In summary, this study establishes a robust prognostic model for OSCC that uncovers the mechanistic link between environmental pollutants—particularly acrolein—and tumor progression.

In summary, this study not only establishes a prognostic model for OSCC based on acrolein- and smoking-related genes, but also uncovers a potential mechanistic link between environmental pollutants—particularly acrolein—and tumor progression. These findings highlight the pivotal role of environmental exposures in shaping cancer risk and tumor biology, offering mechanistic insights into how such pollutants may influence OSCC development and patient prognosis. Importantly, the results carry significant implications for public health and cancer prevention, suggesting that reducing acrolein exposure could be an effective strategy for OSCC risk management. More broadly, this study emphasizes the close interplay between environmental pollutants and human health, reinforcing the necessity of implementing measures to minimize exposure to carcinogenic agents for the protection of population-level health.

## 4. Materials and Methods

### 4.1. Data Collection

Transcriptome profiling data and corresponding clinical annotations for the Head and Neck Squamous Cell Carcinoma (HNSC) project were retrieved from The Cancer Genome Atlas (TCGA) repository (https://www.cancer.gov/ccg/research/genome-sequencing/tcga) (accessed on 2 October 2025) [42]. From this cohort, samples specifically diagnosed as OSCC were isolated for downstream investigation. For external validation purposes, the OSCC microarray dataset GSE41613, including pertinent clinical metadata, was procured from the Gene Expression Omnibus (GEO) database (http://www.ncbi.nlm.nih.gov/geo/) (accessed on 2 October 2025) [43]. This study also utilized the CTD (http://ctdbase.org) (accessed on 2 October 2025), an open resource that curates the effects of toxicants and environmental pollutants on human health. The database encompasses manually curated data characterizing the complex interactions between chemicals and genes/proteins, as well as the associations linking chemicals and genes to specific diseases. Based on this resource, a total of 2608 acrolein-related genes were retrieved, with inclusion restricted to genes derived from human studies.

### 4.2. Identification of Differentially Expressed Genes in OSCC

Raw RNA-seq data from the TCGA dataset were preprocessed using R software (V 4.5.1). For genes with duplicate Gene Symbols, the entry with the highest mRNA expression value was retained. Seventy-three samples with annotation issues were excluded, and genes with read counts <10 in more than 80% of samples were filtered out. Comparing 290 OSCC samples against 40 normal controls, we assessed differential mRNA expression using the DESeq2 algorithm (V 1.48.1) [44]. The criteria for statistical significance were established as an adjusted *p*-value < 0.05 and an absolute |log_2_ fold change| > 2.

The expression matrix was transformed using log_2_ (counts + 1), followed by quartile normalization. Z-score normalization was subsequently applied to the expression values of each mRNA. DEGs between tumor and adjacent normal tissues were identified and visualized using a volcano plot.

### 4.3. Identification of OSCC Intersection Genes Based on Acrolein-Related Genes and GSVA Pathway Enrichment Analysis

The intersection between DEGs identified from the TCGA dataset and the 2608 acrolein-related genes was determined using the Sangerbox online tool and visualized with a Venn diagram [45]. Heatmap visualization of the intersecting genes was performed using the pheatmap R package (V 1.0.13) [46]. GSVA was conducted on the intersection genes using the GSVA R package (V 2.2.0) [47]. Human (Homo sapiens) was selected as the species, and the C2 gene set from the MSigDB database (category = “C2”) was used for annotation. Enrichment scores for each pathway were calculated by GSVA, and differential analysis was performed using the limma package (V 3.64.3) to identify pathways significantly dysregulated between normal and OSCC tissues. GSVA enrichment results were subsequently visualized.

### 4.4. PPI Network Construction and Hub Gene Identification

Protein–protein interaction (PPI) network was constructed utilizing the STRING database (https://string-db.org/) (accessed on 13 October 2025) with a minimum interaction score of 0.4. Subsequently, the network was visualized using Cytoscape software (V 3.10.2). To identify key nodes, the CytoHubba plugin was employed to calculate degree centrality; genes with the highest rankings were designated as hub genes and prioritized for further investigation.

### 4.5. Construction of a Prognostic Risk Model Based on Machine Learning

Prognostic risk models for OSCC were constructed using two machine learning approaches: SVM-RFE and LASSO-Cox regression. To ensure the identification of purely molecular biomarkers, clinical variables were excluded during this feature selection phase. First, the SVM-RFE algorithm was implemented in R using the e1071 package (V 1.7.16) with the following parameters: SVM = rfeControl (functions = caretFuncs, method = “cv”, number = 10, method = “svmLinear”). Ten-fold cross-validation was applied to select the optimal feature set, and features corresponding to the lowest cross-validation error were chosen as candidate biomarkers.

Subsequently, the glmnet R package (V 4.1-8) was used to integrate patient survival time, survival status, and gene expression data for LASSO-Cox regression analysis [48]. To mitigate overfitting through regularization, we implemented LASSO-Cox regression analysis using the glmnet R package. Ten-fold cross-validation was utilized to ascertain the optimal penalty parameter (λ) corresponding to the minimum cross-validation error, thereby ensuring model robustness. Consequently, the individual risk score was derived using the following formula:

$$Risk\;score=\sum_{i=1}^{\infty }\left({coef}_{i}\times {Exp}_{i}\right)$$

where *coef* represents the regression coefficient and Exp represents the gene expression value. The optimal cutoff value for the risk score was determined using the maxstat R package (V 0.7-25), and patients were stratified into high- and low-risk groups accordingly [49]. Survival outcomes were visualized using Kaplan–Meier curves generated via the Survminer package (V 0.4.9), where a log-rank *p*-value of less than 0.05 indicated statistical significance. To further evaluate the predictive accuracy of the risk score, time-dependent ROC curves were constructed utilizing the pROC R package (V 1.18.5). Additionally, the Concordance Index (C-index) was computed to measure the discriminatory capacity of the model [50].

### 4.6. Validation and Evaluation of the Prognostic Risk Model

To validate the robustness of the acrolein-related gene–based OSCC prognostic risk model, the risk score formula derived from the TCGA dataset was applied to the GSE41613 cohort. Gene symbols with duplicate entries were consolidated by averaging their expression values. Subsequently, the expression matrix was subjected to quartile normalization utilizing the limma R package [51]. To delineate differentially expressed genes (DEGs) distinguishing tumor tissues from adjacent normal counterparts, we applied significance criteria of an absolute log_2_ fold change (|log_2_FC|) > 1 and a Benjamini–Hochberg adjusted *p*-value < 0.05. Risk scores were derived for individuals in the validation cohort. To assess the predictive efficacy of the prognostic model, we generated Kaplan–Meier survival plots and receiver operating characteristic (ROC) curves utilizing the Survminer and pROC R packages.

### 4.7. Clinical Characteristics and Pathway Enrichment Analysis of High- and Low-Risk Patients

Based on the TCGA dataset, clinical characteristics of high- and low-risk patients were analyzed in relation to the four key genes, including smoking status, histological type, TNM stage, age, and gender. Statistical significance for prominent clinical variables (specifically, smoking status and pathological stage) was evaluated utilizing the Chi-square test. Furthermore, the correlations between gene expression levels and clinical attributes were depicted via bar plots constructed with the ggplot2 R package (V 4.0.1) [52].

GSVA was subsequently performed on the high- and low-risk groups using the GSVA R package. Differential analysis of pathway enrichment was conducted using the limma package to identify pathways significantly dysregulated between the two risk groups. Differential pathways were visualized to further elucidate the molecular mechanisms and potential functional pathways regulated by acrolein-related DEGs in OSCC across different risk populations.

### 4.8. Immune Infiltration Analysis and Correlation Assessment

The CIBERSORT algorithm was utilized to quantify immune cell infiltration profiles within the TCGA cohort. We determined the relative abundance of distinct immune cell subsets for both high- and low-risk groups, evaluating the interplay among these cells. To visually elucidate cellular interactions, correlation heatmaps were constructed using the corrplot R package (V 0.92) [53]. Furthermore, variations in the abundance of 22 immune cell phenotypes were depicted via boxplots generated with ggplot2. Finally, Spearman correlation analysis, facilitated by the ggstatsplot package (V 0.12.4) [54], was executed to investigate the immunological landscape and its potential prognostic implications in acrolein-associated OSCC.

## 5. Conclusions

This study demonstrates the significant impact of acrolein, a prevalent environmental pollutant, on OSCC development and progression. By analyzing acrolein- and smoking-related genes, we not only established a prognostic model capable of stratifying patients by risk, but also uncovered potential mechanistic links between environmental exposure and tumor biology, including modulation of the immune microenvironment. These findings highlight the pivotal role of environmental pollutants in shaping cancer risk and progression, offering mechanistic insights into how acrolein exposure may influence patient outcomes. Importantly, the results carry substantial implications for public health and cancer prevention, suggesting that reducing acrolein exposure could be an effective strategy for OSCC risk management. More broadly, this study underscores the intimate relationship between environmental toxicants and human health, reinforcing the need for strategies to mitigate exposure to carcinogenic agents and protect population-level health.

## Data Availability

The original data presented in the study are openly available in TCGA (Accession Number: TCGA-HNSC) and GEO (Accession Number: GSE41613) at DOI: 10.1038/nature14129 and DOI: 10.1158/1078-0432.CCR-19-1245.

## Supplementary Materials

The following supporting information can be downloaded at: https://www.mdpi.com/article/10.3390/ijms27020632/s1.
