# Supplementary material for: Transcriptomic Analysis Identifies Acrolein Exposure-Related Pathways and Constructs a Prognostic Model in Oral Squamous Cell Carcinoma
Source: Int J Mol Sci. 2026 Jan 8;27(2):632. doi: 10.3390/ijms27020632 (PMC12841475; doi:10.3390/ijms27020632)
Supplement: Supplementary file 1 [file ijms-27-00632-s001.zip › ijms-4048849-supplementary/Supplement figure.pdf]

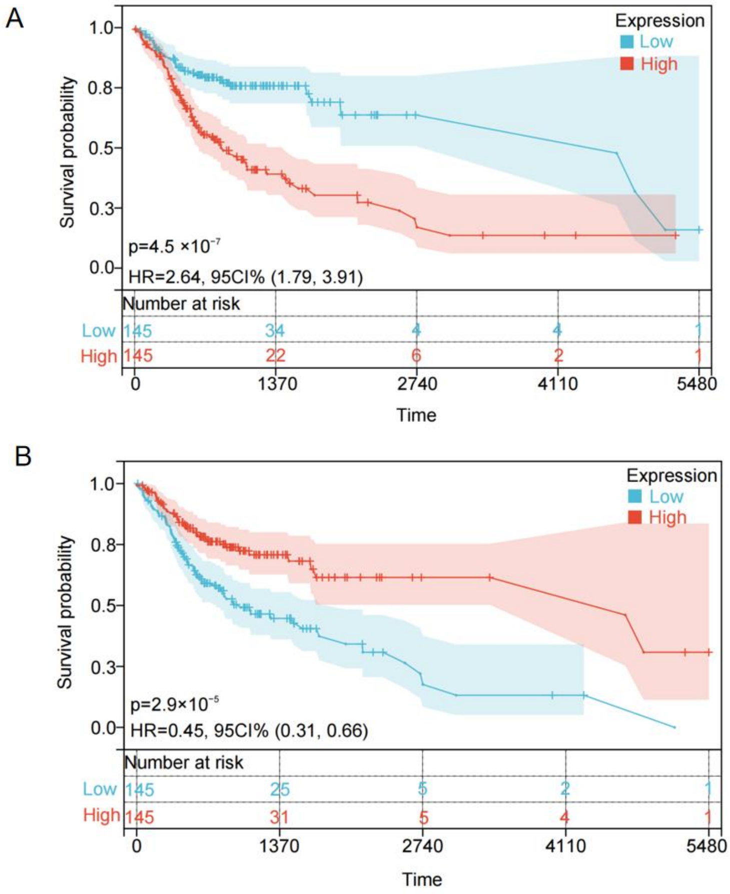

Figure S1. K-M survival analysis of OSCC patients stratified by PPARG and CTLA4 expression. (A) Patients were divided into high and low PPARG expression groups based on the median expression level, and OS was compared; (B) Patients were divided into high and low CTLA4 expression groups, and overall survival was compared.

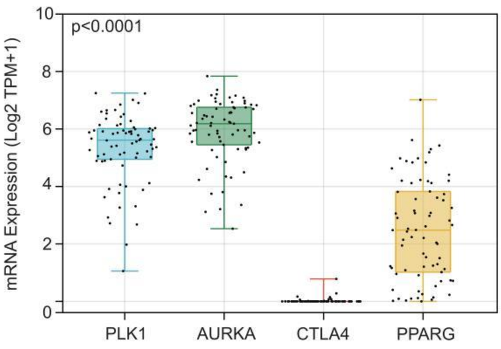

Figure S2. mRNA expression levels of the four signature genes in the malignant tumor cell (single-cell dataset GSE172577) .

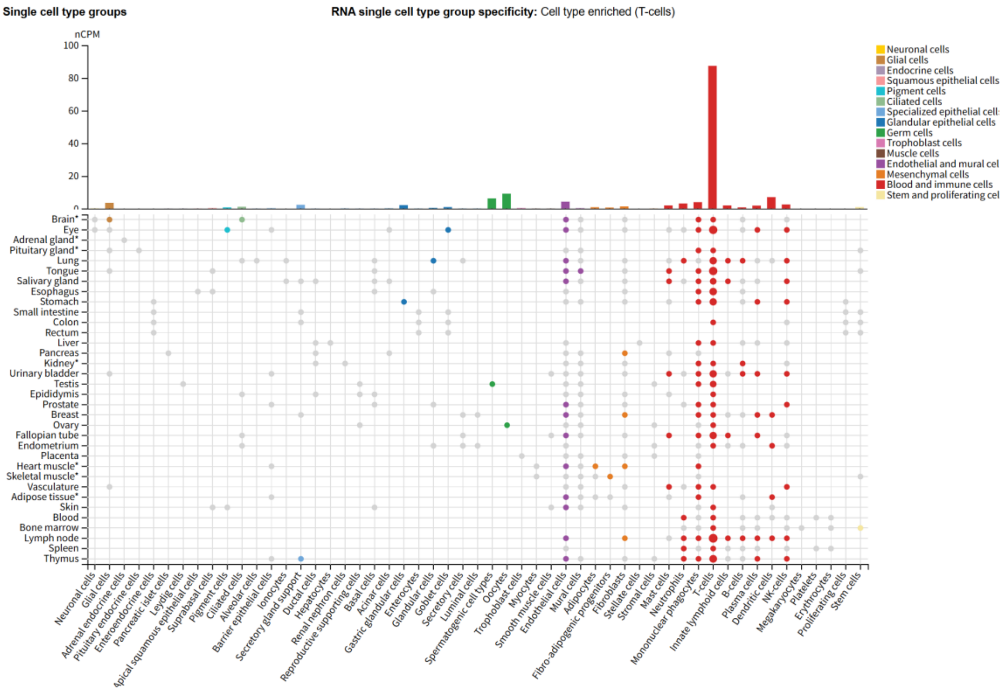

Figure S3. RNA single cell type group specificity of CTLA4. Data retrieved from the Human Protein Atlas classifies CTLA4 as Cell type enriched (T-cells).

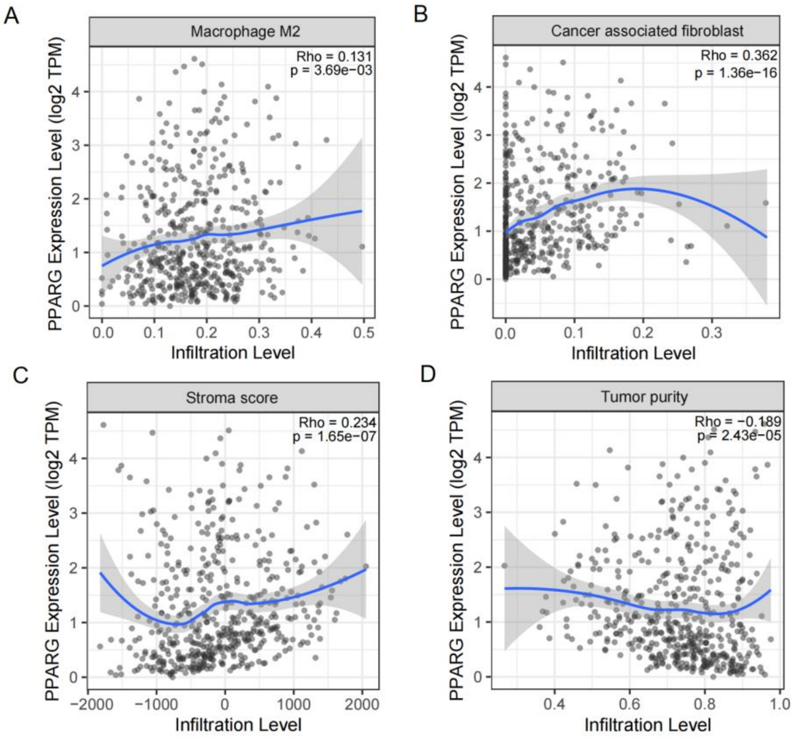

Figure S4. Correlations between PPARG expression and tumor microenvironment characteristics in OSCC. (A) Scatter plot of PPARG expression versus macrophage M2 infiltration level. (B) Scatter plot of

PPARG expression versus cancer associated fibroblast infiltration level. (C) Scatter plot of PPARG expression versus stromal score. (D) Scatter plot of PPARG expression versus tumor purity.

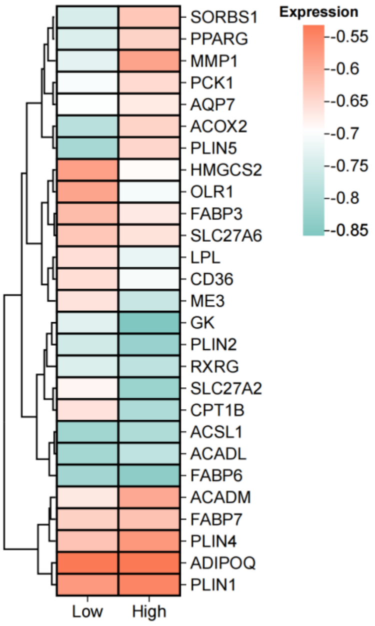

Figure S5. Expression heatmap of key PPAR signaling pathway genes in high- and low-risk groups.

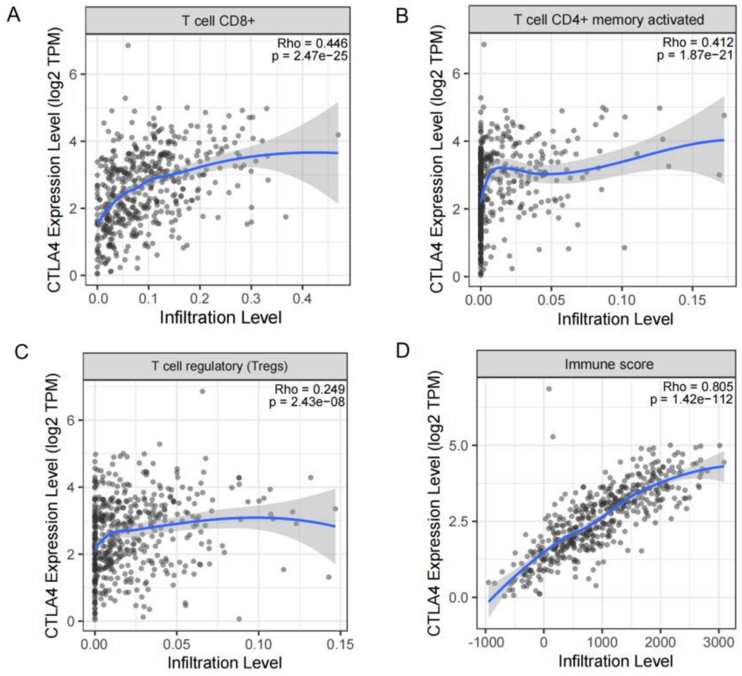

Figure S6. Correlations between CTLA4 expression and tumor microenvironment characteristics in OSCC. (A) Scatter plot of CTLA4 expression versus T cell CD8+ infiltration level. (B) Scatter plot of CTLA4 expression versus T cell CD4+ memory activated infiltration level. (C) Scatter plot of CTLA4 expression versus Tregs. (D) Scatter plot of CTLA4 expression versus immune score.Figure X. Decision

Curve Analysis (DCA) evaluating the clinical utility of the prognostic model for 5-year overall survival.

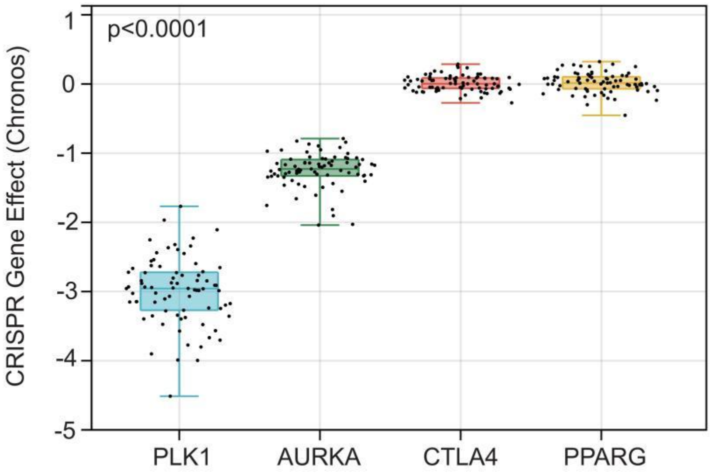

Figure S7. CRISPR-Cas9 gene dependency analysis of the four signature genes retrieved from the DepMap database.

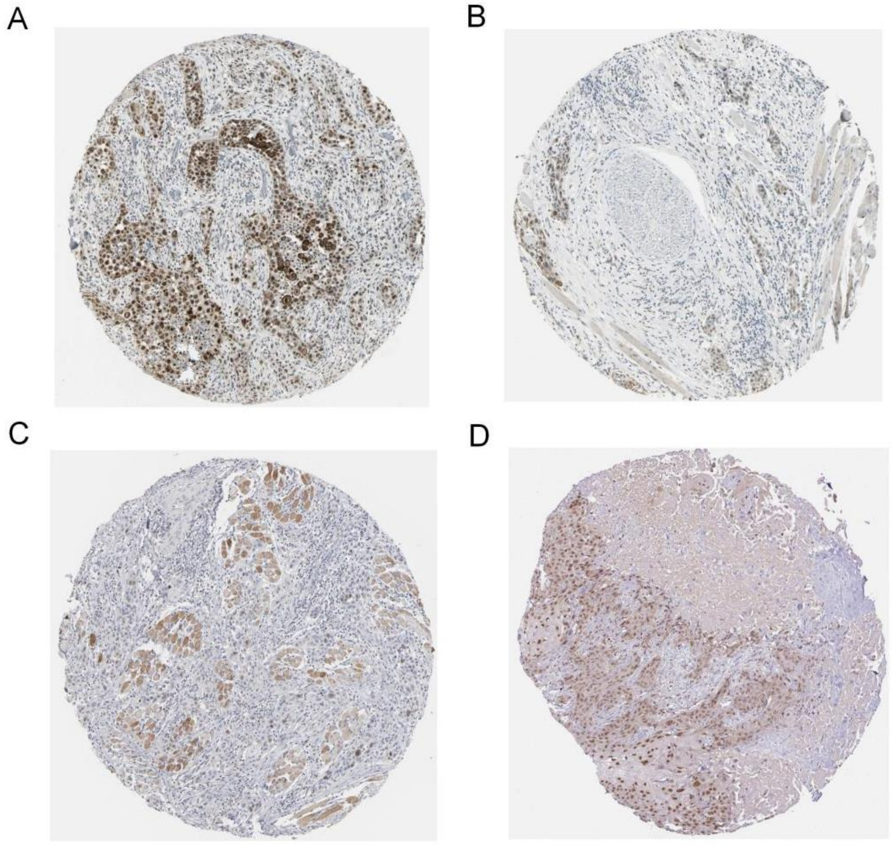

Figure S8. Immunohistochemical (IHC) analysis of the three signature genes. (A)Tumor cells exprese PPARG and are surrounding with CAFs in head and neck squamous cell carcinoma (patient ID: 2624 in HPA); (B) Tumor cells express PPARG and are surrounding with CAFs in

OSCC (patient ID: 1176 in HPA), the tumor tissue is infiltrated with immune cells; (C) Tumor cells express AURKA in OSCC (patient ID: 1176 in HPA); (D) Tumor cells express PLK1 in head and neck squamous cell carcinoma (patient ID: 3360 in HPA).

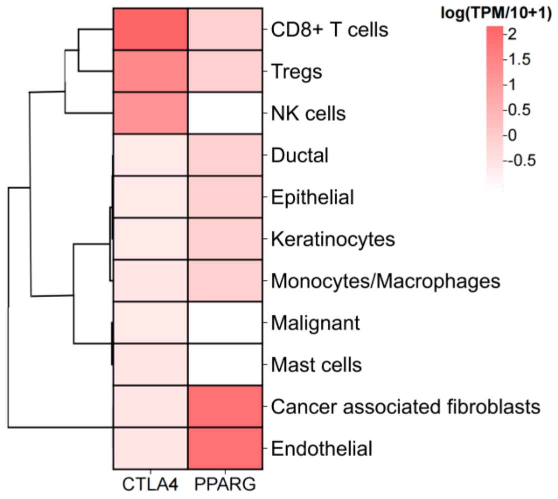

Figure S9. Correlation analysis and immune cell infiltration in the tumor microenvironment.

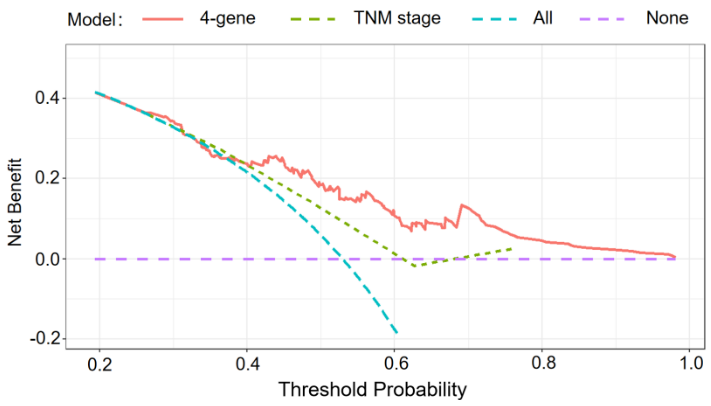

Figure S10. Decision Curve Analysis (DCA) evaluating the clinical utility of the prognostic model for 5-year overall survival.

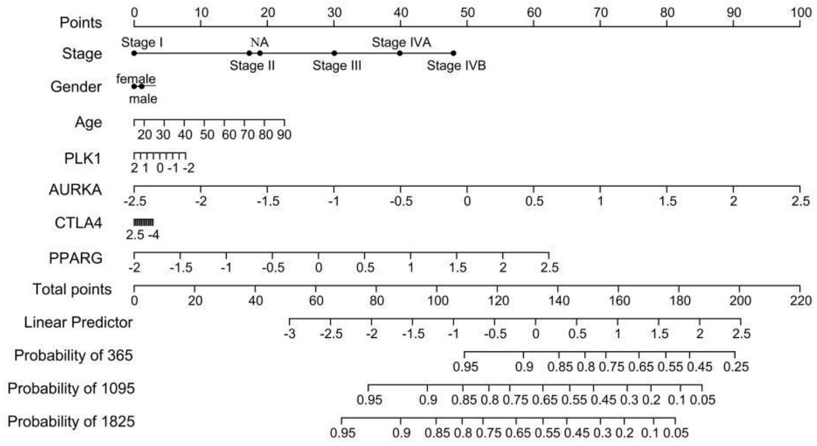

Figure S11. Development of a multivariate nomogram for OSCC prognosis.

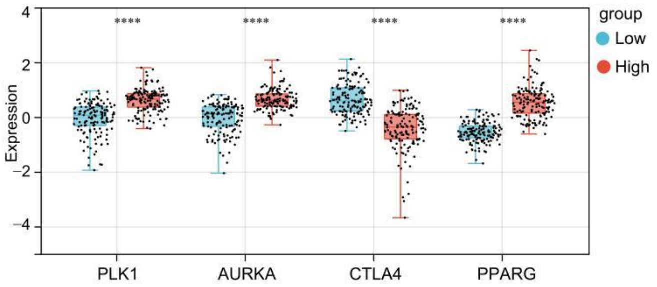

Figure S12. Boxplots showing the expression levels of the four signature genes in the high- and low-risk groups within the TCGA-OSCC cohort.
